# Regulation of CNKSR2 protein stability by the HECT E3 ubiquitin ligase Smurf2, and its role in breast cancer progression

**DOI:** 10.1186/s12885-018-4188-x

**Published:** 2018-03-13

**Authors:** Diana David, Arun Surendran, Jissa V. Thulaseedharan, Asha S. Nair

**Affiliations:** 1 Cancer Research Program, Rajiv Gandhi Centre for Biotechnology, Trivandrum, Kerala 695 014 India; 2 Achutha Menon Centre for Health Science Studies (AMCHSS), Sree Chitra Thirunal Institute for Medical Sciences and Technology, Trivandrum, Kerala 695 011 India; 3 Mass Spectrometry and Proteomics Core Facility, Rajiv Gandhi Centre for Biotechnology, Trivandrum, Kerala 695 014 India

**Keywords:** Smurf2, CNKSR2, Ubiquitination, Breast cancer

## Abstract

**Background:**

Smurf2 E3 ubiquitin ligase physically associates with and regulate the stability of distinct cellular protein substrates. The multi-functional scaffold protein Connector enhancer of kinase suppressor of ras 2 (CNKSR2) plays a key role in regulating cell proliferation, and differentiation through multiple receptor tyrosine kinase pathways. The aim of this study was to investigate whether the interaction between Smurf2 and CNKSR2 has any significant role in the post transcriptional regulation of CNKSR2 expression in breast cancer. Methods**:** Here we demonstrate a novel interaction of CNKSR2 with Smurf2 by co-immunoprecipitation, indirect immunofluorescence studies, and surface plasmon resonance (SPR) analysis, which can ubiquitinate, but stabilize CNKSR2 by protecting it from proteasome mediated degradation.

**Results:**

CNKSR2 protein levels were significantly increased upon forced overexpression of Smurf2, indicating the role of Smurf2 in regulating the stability of CNKSR2. Conversely, Smurf2 knockdown resulted in a marked decrease in the protein level expression of CNKSR2 by facilitating enhanced polyubiquitination and proteasomal degradation and reduced the proliferation and clonogenic survival of MDA-MB-231 breast cancer cell lines. Tissue microarray data from 84 patients with various stages of mammary carcinoma, including (in order of increasing malignant potential) normal, usual hyperplasia, fibrocystic changes, fibroadenoma, carcinoma-in-situ, and invasive ductal carcinoma showed a statistically significant association between Smurf2 and CNKSR2 expression, which is also well correlated with the ER, PR, and HER2 status of the tissue samples. A comparatively high expression of Smurf2 and CNKSR2 was observed when the expression of ER and PR was low, and HER2 was high. Consistently, both Smurf2 and CNKSR2 showed an integrated expression in MCF10 breast progression model cell lines.

**Conclusions:**

Altogether, our findings reveal that Smurf2 is a novel positive regulator of CNKSR2 and suggest that Smurf2-CNKSR2 interaction may serve as a common strategy to control proliferation of human breast cancer cells by modulating CNKSR2 protein stability.

**Electronic supplementary material:**

The online version of this article (10.1186/s12885-018-4188-x) contains supplementary material, which is available to authorized users.

## Background

The ubiquitin-proteasome system (UPS) plays a prominent role in cell regulatory mechanism to maintain homeostasis. Evidently, every part of the cell is under the control of the ubiquitin-proteasome regulatory system. The peptide products of this proteasome system provides additional outcome by functioning as a critical factor in deciding whether a cell will be recognized as infected or tumorigenic by the immune system and destroyed, or recognized as self and spared [1]. In many cases, stability of several important scaffold proteins is controlled by ubiquitination, a predominant posttranslational modification central to eukaryotic regulation [2, 3]. E3s carry out the key rate-limiting step in ubiquitin-mediated proteolysis. Multiple E3 ubiquitin ligases can targets same protein and vice versa, corroborating a highly complex and dynamic regulation [4]. Hence, the dynamic role of cellular ubiquitination can be further confirmed by identifying new targets for a given ubiquitin ligase and, conversely, new ubiquitin ligases for a given target [4].

Smurf2 is a C2-WW-HECT E3 ubiquitin ligase that has the ability to ubiquitinate and degrade some proteins, e.g., the TGF-β receptor I and associated Smad proteins involved in TGF- β/BMP signaling, but also have a unique ability to protect other substrates, e.g., spindle assembly checkpoint protein, MAD2 and NEDD9/HEF1 [5]. It contains WW domains, which directly bind to a PP*X*Y motif (also known as PY motif) in its targets which is further stabilized by the PY tail, a six-amino acid stretch immediately carboxylterminal to the PP*X*Y motif, although additional interactions exist [6]. Smurfs are frequently deregulated in human cancers and are critical regulators of multiple oncogenic factors. However, the specific role of Smurf2 in tumorigenesis has been highly obscure, with numerous studies showing that it is either pro-tumorigenic or, conversely, anti-tumorigenic in a context-dependent manner. Smurf2 has involved in diverse signal pathways and cellular processes by targeting broad spectrum of proteins. Recent studies emphasize the growing appreciation that the pleiotropic effects of Smurf2 might be explained by the ability of Smurf2 to target potential molecular substrates in a ligase dependent and independent manner. Thus, expanding the family of Smurf2 targets may provide further insights into regulatory role of Smurf E3 ubiquitin ligases in the cellular environment [5].

CNK (Connector enhancer of kinase suppressor of ras) is a putative multi-adaptor scaffold protein required in multiple receptor tyrosine kinase pathways particularly in RAS-RAF/ mitogen–activated protein kinase (MAPK) cascade which is involved in conveying several proliferative and differentiative signals to the nucleus [7]. CNKSR2 (Connector enhancer of kinase suppressor of ras 2) also named as Membrane-Associated GUanylate kinase Interacting protein (MAGUIN) is a mammalian isoform of CNK, and is the closest homolog to *Drosophila melanogaster* and *C. elegans* CNK, thus likely represents the orthologous member. The neuronal isoform CNK2 has an essential function in nerve growth factor-induced, sustained stimulation of ERK leading to neuronal differentiation [8]. But CNK2 is not required for epidermal growth factor-dependent, transient stimulation of ERK occurring during cell proliferation. CNK’s multidomain architecture suggests that it has the ability to bind and bring together different molecules as previously shown for several other multidomain molecules [8]. In particular, consistent with other known potential substrates of Smurf2, we have also identified a probable Smurf2-interacting motif in CNKSR2, a ‘SPPPPY’ motif at 702–707 sequence region that shows a strong PY motif match with Smurf2. Indeed, we observed that knockdown of Smurf2 downregulated the expression of CNKSR2 and reduced the proliferative potential of human breast cancer cells [9]. Hence we hypothesized that CNKSR2 may be a novel substrate for Smurf2 E3 ubiquitin ligase which seems to perform a crucial role in regulating the stability of CNKSR2. However, whether additional ubiquitin ligases for CNKSR2 exist and how CNKSR2 is regulated by various ubiquitin ligases are not clearly defined.

## Methods

### Plasmids, cell lines and culture conditions

pCMV5B-Flag-Smurf2 and pCMV5B-Flag-Smurf2 C716A plasmids have been described [10] and were purchased from Addgene, Cambridge, MA, USA. Human embryonic kidney 293 (HEK293) cells were purchased from National Centre for Cell Science (NCCS, Pune, India). Immortalized normal human breast epithelial cell line MCF10A, and the human breast cancer MCF-7 and MDA-MB-231 cells were purchased from the American Type Culture Collection (ATCC; Manassas, VA, USA). The pre-malignant and in situ grade cell lines-MCF10AT and MCFDCIS were a kind gift from Dr. Suresh Kumar Rayala (Indian Institute of Technology-Madras, India). MCF10A and MCF10AT cells were cultured in Dulbecco’s modified eagles medium (DMEM)/Nutrient F12-Ham (1:1) supplemented with 10% fetal bovine serum (FBS), 20 ng/ml Epidermal Growth Factor (EGF), 100 ng/ml cholera toxin, 0.01 mg/ml Insulin-Transferrin-Selenium (ITS), 500 ng/ml hydrocortisone, and 1 ng/ml Fibroblast Growth Factor (FGF). MCFDCIS, MCF-7 and MDA-MB-231 cells were grown at 37 °C with 5% CO_2_ in Dulbecco’s modified eagles medium (DMEM) medium supplemented with 10% FBS and 1% penicillin/streptomycin (Invitrogen; Carlsband, CA, USA).

### Tissue samples

Human breast tissue samples were collected from Regional Cancer Centre (RCC), Thiruvananthapuram, India after obtaining approval from the Institutional Human Ethics Committee. The samples were collected from patients who underwent primary surgery without prior chemotherapy or radiotherapy. Informed consent was taken from all the patients prior surgery/excision of tissue.

### siRNA and shRNA transfections

siRNA against Smurf2, in the form of either a mixture of three siRNAs targeting different regions of Smurf2 (Santa Cruz Biotechnology; Texas, USA), or the negative control siRNA included in the kit (Santa Cruz Biotechnology; Texas, USA) was transfected into 60–70% confluent MDA-MB-231 cells with siLentFect (Bio-Rad; CA, USA) according to the manufacturer’s instructions. Three days later, cells were subjected to western blotting, immunoprecipitation, and quantitative RT-PCR analysis.

Stable knockdown of Smurf2 in MDA-MB-231 cells was achieved by transfecting Smurf2 shRNA plasmid containing a pool of three target-specific lentiviral vector plasmids each encoding 19–25 nucleotide (plus hairpin) shRNAs designed to knockdown Smurf2 gene expression (Santa cruz biotechnology; Texas, USA) using Amaxa® Cell Line Nucleofector® Kit V (Lonza; Basel, Switzerland) according to the manufacturer’s instructions. Briefly, 1 × 10^6^ cells were transfected with 2 μg plasmid DNA in 100 μl cell Line Nucleofector® Solution V (Lonza; Basel, Switzerland) and seeded in 6-well plate containing pre-equilibrated DMEM medium. After 24 h, cells were selected for stable integration using 1 μg/ml Puromycin (Sigma; St. Louis, MO, USA) for 3 weeks and were used for further experiments. MDA-MB-231 cells stably expressing Control shRNA Plasmid-A (Santa Cruz Biotechnology; Texas, USA) were also generated in the same fashion to serve as negative control.

### Immunoprecipitation

For cultured cells, in a 10-cm dish format, HEK293 cells transfected with empty vector, pCMV5B-Flag-Smurf2 WT, or pCMV5B-Flag-Smurf2 C716A were washed in 1 × PBS and resuspended in 1 ml of lysis buffer (70 mM NaCl, 50 mM Tris, pH 8 and 0.5% NP-40), supplemented with phosphatase inhibitor and protease inhibitor cocktails (Sigma; St. Louis, MO, USA). After rotating at 4 °C for 30 min, the cell lysate was collected and precleared by spinning at 14,000 rpm for 10 min. For co-immunoprecipitation of endogenous Smurf2 with CNKSR2, total protein was isolated from MDA-MB-231 as described previously. For each pull down, 3 μg of corresponding antibody was added to the normalized lysate (1 mg of total protein by Bradford’s Assay, Bio- Rad; CA, USA)) and the mixture was incubated overnight at 4 °C. Normal rabbit IgG was used as negative control. Immune complexes were then precipitated with protein A sepharose macrobeads (Sigma; St. Louis, MO, USA). Beads were washed four times with lysis buffer and immunoprecipitated samples were resolved by SDS-PAGE for immunoblotting.

### Surface plasmon resonance (SPR)

Binding kinetics was determined by SPR using a ProteOn XPR36™ SPR instrument (Bio-Rad; CA, USA) with a GLC (General Layer Compact) biosensor chip (Bio-Rad; CA, USA) with its unique “one-shot” kinetics approach. The apparatus has six parallel flow channels for uniformly immobilizing strips of six ligands on the sensor surface. The fluidic system can be rotated automatically to 90° so that up to six different analytes can be injected, thereby simultaneously monitoring up to 36 individual molecular interactions in a single run on a single chip [11]. In our study, the ligand (recombinant Smurf2 protein; Sigma; St. Louis, MO, USA) was immobilised to the biosensor surface layer by amine coupling chemistry, followed by capture of the analyte (recombinant CNKRS2 protein; Cusabio Biotech, China) as described previously (Bravman et al., [11]). One of the six ligand channel (L4) was used to capture the Smurf2 protein and one ligand channel was left blank (L6) by injecting the 10 mM sodium acetate buffer, pH 4.0 (without Smurf2), which served as the ligand reference channel. The chip surface was activated for the two ligand channels (L4 and L6) using a mixture of 5 mM N-hydroxysulfosuccinimide (sulfo-NHS) and 20 mM 1-ethyl-3(3-dimethylaminopropyl)-carbodiimide (EDC), ethanolamine-HCl) mixed in 1:1 ratio to maintain a uniform composition throughout the chip. This was followed by an immediate injection of 300 μl of 7.5 μg/ml of the recombinant Smurf2 protein in 10 mM sodium acetate buffer, pH 4.0 for 600 s. Immobilisation was performed at 25 °C at a flow rate of 30 μl/min using PBS/Tween (Phosphate buffered saline, pH 7.4, 0.005% Tween 20), as the running buffer. The sensor chip surface was then inactivated using 120 μl of 1 M ethanolamine-HCl to block residual NHS-activated carboxyl groups on the chip surface. The final immobilization levels of the Smurf2 ligand was ~ 2000 response units (RU) (1 RU = 1 pg of protein/mm^2^).

During the interaction step the running buffer used was 20 mM Tris-HCl, pH -8.0, 0.5 M NaCl. The system was primed with the running buffer before the start of the experiment. Four of the six analyte channels (A1-A4) were used to capture CNKRS2 at four different concentrations using a twofold dilution series ranging from 0.25 μM to 2 μM. All the four different analyte concentrations were passed simultaneously (a single injection) in the analyte direction at a flow rate of 100 μl/min for 60 s of association with Smurf2, followed by dissociation for 600 s. In the sixth channel (A6), ‘analyte running buffer’ without any protein was injected for double referencing. All these assays were done at 25 °C. All binding sensorgrams were collected, processed, and analyzed using the integrated ProteOn Manager software version 3.1.0.6 (Bio-Rad; CA, USA). Response data from the ProteOn instrument were normalized to a baseline value of zero just prior to the start of the analyte injection. Responses from the reference positions before and after each reaction spot were corrected by subtracting the non-specific response observed in the reference surface to remove non-specific binding and the large step changes in response due to changes in buffer refractive index (‘buffer shift’). Finally, the binding curves were grouped fitted using the Langmuir model describing 1:1 binding stoichiometry with one local parameter for surface capacity (R_max_), one association rate constant (k_a_ or k_on_), and one dissociation rate constant (k_d_ or k_off_). The ratio of the rate constants (k_d_/k_a_) yielded the value for the equilibrium or affinity constant (K_D_).

### Immunofluorescence microscopy

HEK293 cells were transfected with pCMV5B-Flag-Smurf2 WT, fixed with ice-cold acetone/methanol (1:1) and blocked with 3% BSA in PBS for 1 h at room temperature. Cells were then incubated with mouse Flag (Sigma; St. Louis, MO, USA) and rabbit CNKSR2 (Abcam; Cambridge, UK) antibodies, followed by incubation with FITC-conjugated anti-mouse and PE-conjugated anti-rabbit (Santa cruz Biotechnology; Texas, USA) secondary antibodies. For immunofluorescence of endogenous Smurf2 and CNKSR2, cells were fixed with ice-cold acetone/methanol (1:1) and blocked with 3% BSA in PBS for 1 h at room temperature. Cells were then incubated with goat Smurf2 (Santa Cruz Biotechnology; Texas, USA) and rabbit CNKSR2 (Abcam; Cambridge, UK) antibodies, followed by incubation with FITC-conjugated anti-goat and PE-conjugated anti-rabbit (Santa Cruz Biotechnology; Texas, USA) secondary antibodies. Cells were also stained with Propidium Iodide (PI) and Hoecht dye to reveal nuclei. Immunofluorescence was monitored by both conventional and confocal microscopy.

### Ubiquitination assay

For ubiquitination of endogenous CNKSR2 after Smurf2 overexpression, HEK293 cells were transfected with Smurf2WT, Smurf2C716A, and empty vector and disrupted in IP lysis buffer (70 mM NaCl, 50 mM Tris, pH 8, 0.5% NP-40) supplemented with 1 × phosphatase inhibitor and 1 × protease inhibitor cocktails (Sigma; St. Louis, MO, USA). Lysates were denatured by boiling for 15 min and diluted 10-fold in IP buffer (70 mM NaCl, 50 mM Tris, pH 8, 0.5% NP-40) supplemented with 1 × phosphatase inhibitor and 1 × protease inhibitor cocktails (Sigma; St. Louis, MO, USA). The lysates were immunoprecipitated with rabbit CNKSR2 (Abcam; Cambridge, UK) followed by incubation with protein A sepharose macrobeads (Sigma; St. Louis, MO, USA). Ubiquitinated CNKSR2 was revealed by mouse ubiquitin (Santa Cruz Biotechnology; Texas, USA) immunoblotting. 1% total cell lysate was also included as Input control.

For ubiquitination of endogenous CNKSR2 after Smurf2 knockdown, MDA-MB-231 cells were seeded at 2 × 10^5^ cells/well in 6-well format and treated with Control siRNA or Smurf2-specific siRNA. Cells were treated for 4 h with 10 μM MG132 (Calbiochem; CA, USA) 44 h post-transfection, and disrupted in the lysis buffer (70 mM NaCl, 50 mM Tris, pH 8 and 0.5% NP-40). Lysates were denatured by boiling for 15 min and diluted 10-fold in IP buffer (70 mM NaCl, 50 mM Tris, pH 8, 0.5% NP-40 1Xprotease inhibitor, 1XSer/Thr and Tyr phosphatase inhibitor). The lysates were immunoprecipitated with either control rabbit IgG (Santa cruz biotechnology; Texas, USA), or rabbit CNKSR2 (Abcam; Cambridge, UK). Entire immunoprecipitates were loaded onto gel; gel was transferred onto PVDF membrane as usual. Ubiquitinated CNKSR2 was revealed by mouse ubiquitin (Santa Cruz Biotechnology; Texas, USA) immunoblotting. 1% total cell lysate was also included as Input control.

### Immunohistochemistry

Breast cancer tissue arrays with progressive changes- BRC961 and BRC962, non-overlapping with each other and each containing 48 cases from normal, premalignant and cancer tissues with progressive grades and stages in duplicates were purchased from Pantomics. Expression of Smurf2 and CNKSR2 was evaluated using an indirect immunoperoxidase procedure (ABC-Elite, Vector Laboratories; CA, USA). Briefly, TMA slides were baked at 60 °C for 30 min before use. The slides were then deparaffinized in xylene and rehydrated in graded ethanol/isopropanol. Endogenous peroxidase activity was blocked by incubating the slides in 1.5% H_2_O_2_ in methanol for 20 min at room temperature. Antigen retrieval was done by dipping the slides for 12 min in boiling citrate buffer*.* The slides were then blocked with 3% BSA-PBS for 1 h at room temperature. After blocking, the sections were incubated with primary antibodies; rabbit Smurf2 (Santa cruz Biotechnology; Texas, USA) and rabbit CNKSR2 (Abcam; Cambridge, UK), in 1% BSA-PBS at 4 °C overnight. Bound antibody was detected using Vectastain ABC kit (ABC-Elite, Vector Laboratories; CA, USA). Briefly, post PBS wash, the sections were incubated with universal biotinylated anti-mouse IgG/rabbit IgG secondary antibody (C) in normal horse serum blocker (supplied in the kit) for 30 min at room temperature. The sections were then washed thrice in 1XPBS followed by incubation with A + B mixture (Avidin+Biotinylated Horseradish Peroxidase) for another half an hour at room temperature. The sections were again washed with 1XPBS and incubated in DAB (Sigma; St. Louis, MO, USA) solution till they developed brown color. Further, the sections were washed in distilled water and counterstained with hematoxylin for 1 min for nuclear staining. The sections, after bluing for 10 min under running tap water were dehydrated in graded ethanol changes and finally, cleared in xylene. The sections were then mounted using DPX (Qualigens; India) and visualized using a digital up-right microscope, Leica DMI 1000, at 40× magnification.

For scoring, at least five randomly selected high power fields were chosen and a total number of 1000 cells were considered. Only epithelial cells were evaluated. The cells were considered nuclear positive if the nuclei stained positive irrespective of the intensity of staining. Cytoplasmic positivity of Smurf2 and CNKSR2 were measured depending on the intensity of immunoreactivity and scored as mild (+), moderate (++), and intense (+++). The staining intensity in each core biopsy was recorded separately. Slides were read blinded by two readers (D.D. and A.N.) along with discussions made with an expert pathologist. The IHC data regarding ER, PR, and HER2 staining intensity of the corresponding tissues were obtained from Pantomics.

### Quantitative real time PCR

shRNAs against Smurf2 (Santa Cruz Biotechnology; Texas, USA) or the negative control shRNA (Santa cruz biotechnology; Texas, USA) was transfected into MDA-MB-231 cells using Amaxa® Cell Line Nucleofector® Kit V (Lonza; Basel, Switzerland) according to the manufacturer’s instructions. Cells were selected for stable integration using 1 μg/ml Puromycin (Sigma; St. Louis, MO, USA) for 3 weeks and total RNA was isolated with TRIzol (Ambion/Invitrogen; Carlsband, CA, USA), and quantitative real-time RT-PCR was performed in three triplicates with primer sets specific for Smurf2(forward,TGGATCAGGAAGTCGGAAAA and reverse, GGACATGTCTAACCCCGGA) and CNKSR2 (forward, CGACCTCCCTCGATGAGTTG and reverse, CACTGCACTGCTCCCAGTTA) and the control gene GAPDH (forward, TTGGTATCGTGGAAGGACTCA and reverse, TGTCATCATATTTGGCAGGTT). Products were amplified and detected with the Power SYBR Green PCR Master Mix (Applied Biosystems; Massachusetts, USA) on an.

ABI 7900HT Fast Real-Time PCR System (Applied Biosystems; Massachusetts, USA) according to the manufacturer’s instructions. The real time analyses were done by 2^-ΔΔCt^ method [12] using SDS 2.1 software (Applied Biosystems; Massachusetts, USA) after normalization with the GAPDH control.

### Clonogenic assay

Clonogenic assay or foci formation assay was performed as described previously [13]. Briefly, cells were seeded at a density of 500 cells/well in six-well plates and incubated for 14 days with intermittent media change at every 2 days. After 14 days, plates were rinsed carefully twice with 1 × PBS and fixed with 4% paraformaldehyde. After fixation, cells were washed thrice with 1 × PBS and stained with 0.5% crystal violet for 30 min at room temperature on a rocking platform. The dishes were rinsed three times with water, air-dried and analyzed. Colonies comprising more than 50 cells were counted manually and images were obtained using a digital camera. The experiments were done in duplicate at least three times.

### Soft agar colony assay

Anchorage-independent growth was determined by soft agar analysis as follows: Smurf2 knockdown MDA-MB-231 cells were trypsinized and 1 × 10^4^ cells per 35-mm dish were seeded in 0.35% agar on top of a base layer containing 0.8% agar. Plates were incubated at 37 °C at 5% CO_2_ in a humidified incubator for 21 days. After 21 days, the colonies were fixed with 4% paraformaldehyde and stained with 0.05% crystal violet for 1 h. Colonies with > 50 cells were counted under a microscopic field at 10× magnifications. The experiments were done in duplicates at least three times.

### Statistical analysis

Statistical analysis was performed using Intercooled Stata software (Intercooled Stata 11.2 version). The expression of Smurf2, CNKSR2, ER, PR, and HER2 were compared between tumor grades using Chi squared test. The effects of Smurf2 and CNKSR2 expression on histopathological grade of the tumor were estimated with Odds Ratio (OR) (unadjusted Odds ratio) and their 95% Confidence Interval (CI) derived from binary logistic regression model. In order to assess the independent effect of Smurf2, CNKSR2, ER, PR, and HER2 expression on tumor grade, we included all the five factors in the multiple logistic regression model and estimated the Odds Ratio (OR) (adjusted Odds ratio) and their 95% Confidence Interval (CI). All data were obtained by at least three independent experiments and represented as mean ± standard deviation. The limit of statistical significance was set at *P* < 0.05.

## Results

### CNKSR2 is a novel interacting protein of Smurf2

**Fig. 1:**
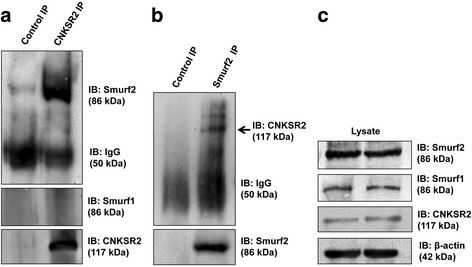
Smurf2 interacts with CNKSR2. **(a**, **b**) CNKSR2 coimmunoprecipitates with endogenous Smurf2 but not Smurf1. Cell lysates were prepared from MDA-MB-231 cells and were immunoprecipitated (IP) with rabbit CNKSR2, rabbit Smurf2 or control antibodies. Precipitates were resolved by 4–10% SDS-PAGE followed by immunoblotting (IB) with rabbit Smurf2, rabbit Smurf1 and rabbit CNKSR2. **c** Protein levels of Smurf2, Smurf1, CNKSR2 and β-actin in 1% of the total cell lysate were also shown

Our previous findings that CNKSR2 encompasses a ‘SPPPPY’ motif at 702–707 amino acid sequence region that forms an energy stable complex with Smurf2 WW2 domain in silico*,* and the decreased expression of CNKSR2 following Smurf2 knockdown [9], prompted us to examine whether there were direct physical and molecular interactions between Smurf2 and CNKSR2. To determine whether endogenous Smurf2 and CNKSR2 interact with each other, we performed co-immunoprecipitation of Smurf2 with CNKSR2 from cell lysates isolated from MDA-MB-231 cells. Indeed, Smurf2 was readily detected in CNKSR2 immunoprecipitates (Fig. 1a) and reciprocally, CNKSR2 was found in Smurf2 immunoprecipitates (Fig. 1b). This interaction is specific for Smurf2, as the interaction of endogenous Smurf1 with CNKSR2 was not detected (Fig. 1a). Thus, CNKSR2 specifically interacts with Smurf2. Our observations do not exclude the possibility that this interaction could require other proteins, but these data do suggest that Smurf2 and CNKSR2 are in complex with each other.

### Smurf2 interacts with CNKSR2 in a ligase independent manner

**Fig. 2:**
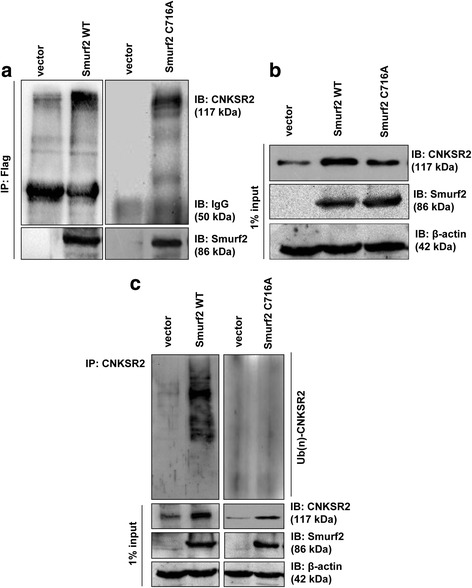
Forced expression of wild-type or catalytically inactive Smurf2 stabilizes CNKSR2 protein. **a** Co-transfection of wild-type (WT) or catalytically inactive (C716A) Smurf2 increased CNKSR2 expression. Smurf2 WT or mutant (C716A), or empty vector, was transfected into HEK293 cells, respectively. 48 h post-transfection, cells were harvested and immunoprecipitated (IP) by anti-Flag antibody followed by immunoblotting (IB) with rabbit CNKSR2 and mouse Flag (for Flag-Smurf2) antibodies. **b** Protein levels of CNKSR2, Flag-Smurf2, and β-actin in 1% of the total cell lysate were also shown. **c** Status of cellular CNKSR2 ubiquitination in Smurf2WT and Smurf2C716A transfected cells. HEK293 cells were transfected with Smurf2WT, Smurf2C716A, or empty vector respectively. After 48 h, cell lysates were harvested, denatured, and subjected to anti-CNKSR2 immunoprecipitation (IP). Precipitates were then resolved by 4–12% SDS-PAGE and immunoblotted (IB) with anti-ubiquitin antibody. CNKSR2, Flag-Smurf2 and β-actin levels were also shown by immunoblotting in 1% input

To confirm that endogenous CNKSR2 associates with Smurf2 and to analyse if forced overexpression of Smurf2 increased CNKSR2 levels, HEK293 cells were transfected with Flag-tagged Smurf2 WT, Flag-tagged Smurf2 C716A, and empty vector followed by immunoprecipitation and immunoblotting. CNKSR2 was identified only in Smurf2-expressing cells (Fig. 2a), and the levels of CNKSR2 were substantially increased by transfection with Flag-tagged Smurf2 WT, compared with control plasmid (Fig. 2b). As Smurf2 is an E3 ubiquitin ligase, we next asked if the Smurf2 enzymatic activity was required for it to interact with CNKSR2. Interestingly, expression of a catalytically inactive Smurf2 mutant (C716A, targeting the HECT domain) also upregulated the levels of CNKSR2 (Fig. 2a, b). These results provide support for the idea that Smurf2 positively regulates CNKSR2, and the Smurf2-CNKSR2 interaction is also independent of the enzymatic activity of Smurf2.

Even though CNKSR2 was found to interact with both wild type and ligase deficient mutant Smurf2, the expression level of CNKSR2 was significantly high in Smurf2 WT transfected cells compared with Smurf2 C716A transfected cells, which prompted us to investigate the ubiquitination status of CNKSR2 following interaction with Smurf2. Hence we immunoprecipitated CNKSR2 from HEK293 cells transfected with either catalytically active wild-type (WT) or a catalytically dead C716A mutant of Smurf2 and immunoblot analysis was performed using mouse ubiquitin antibody. As shown in Fig. 2c, Smurf2 WT moderately increased the ubiquitination of CNKSR2, whereas Smurf2 (C716A) mutant had no effect, indicating that Smurf2 has some role in regulating the ubiquitination of CNKSR2. Altogether, these data indicate that Smurf2 can interact with CNKSR2 leading to its ubiquitination; however, Smurf2-dependent ubiquitination is protective for CNKSR2, which was further supported by an increased expression of CNKSR2 in Smurf2 WT transfected cells compared with Smurf2C716A transfected cells as shown in Fig. 2b.

### CNKSR2 co-localizes with Smurf2

**Fig. 3:**
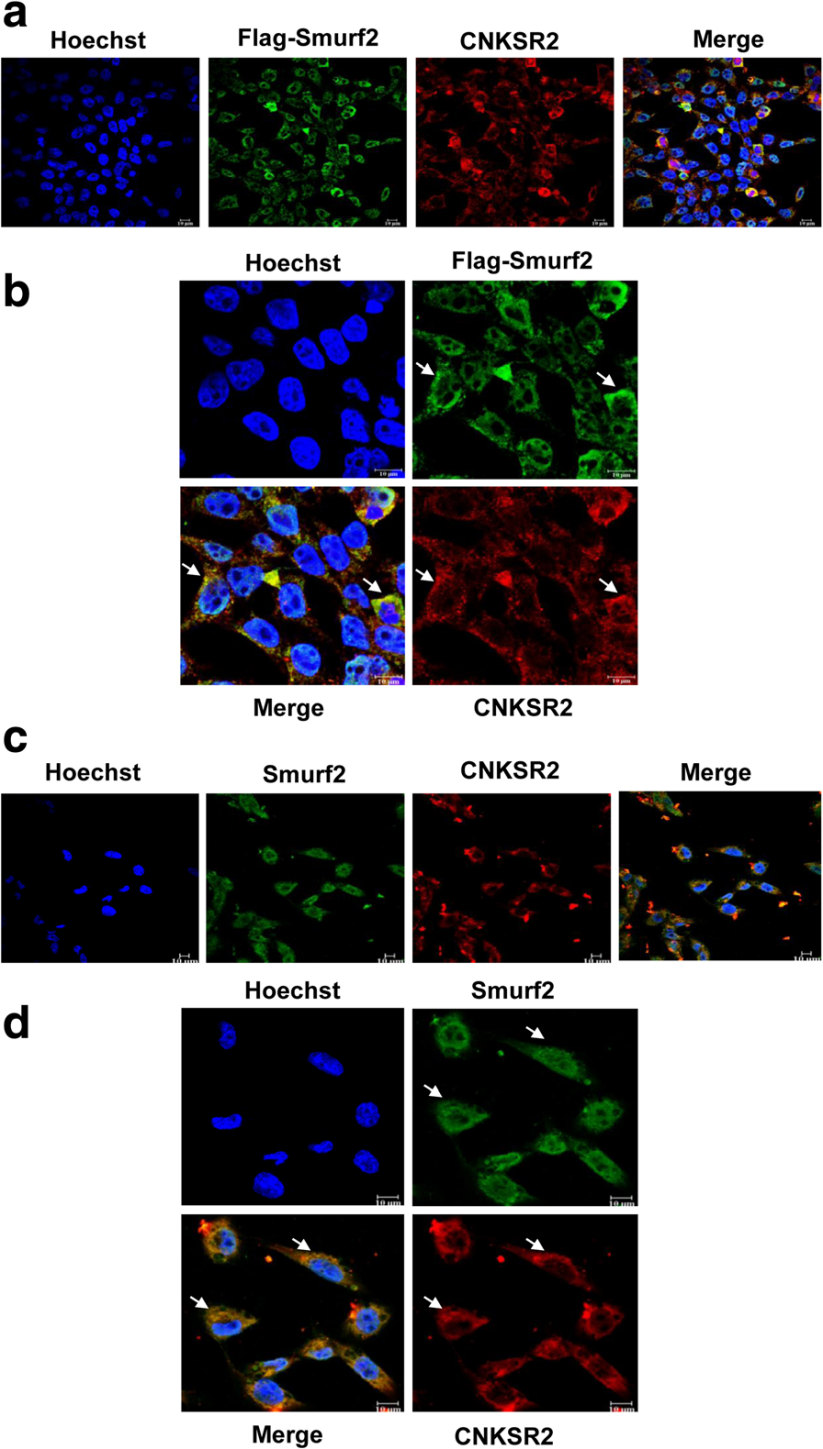
Co-localization of Smurf2 and CNKSR2 in HEK293 and MDA-MB-231 cell lines. **a** HEK293 cells were transfected with Flag-Smurf2, and stained with mouse anti-Flag and rabbit anti-CNKSR2, followed by FITC-conjugated goat anti-mouse and PE-conjugated goat anti-rabbit secondary antibodies. **b** Magnified view of several cells imaged in A. **c** Endogenous Smurf2 and CNKSR2 co-localizes in MDA-MB-231 cells stained with goat anti-Smurf2 and rabbit anti-CNKSR2, followed by FITC-conjugated rabbit anti-goat and PE-conjugated goat anti-rabbit secondary antibodies. **d** Magnified view of several cells imaged in (**c**). Arrows illustrates the presence of co-localization

Consistent with the interaction between Smurf2 and CNKSR2, the two proteins co-localize particularly in the cytoplasm. After HEK293 cells were transfected with Flag-Smurf2, indirect immunofluorescence demonstrated that both CNKSR2 and Smurf2 were primarily co-localized to the cytoplasm (Fig. 3a, b). Endogenous CNKSR2 and Smurf2 also co-localized primarily in the cytoplasm of MDA-MB-231 cells (Fig. 3c, d), and the co-localization was especially visible under higher magnification and better resolution. Combining the results of all the experiments, CNKSR2 apparently interacts with Smurf2 in mammalian cells.

### Binding kinetics of Smurf2 and CNKSR2

#### Capture of Smurf2

**Fig. 4:**
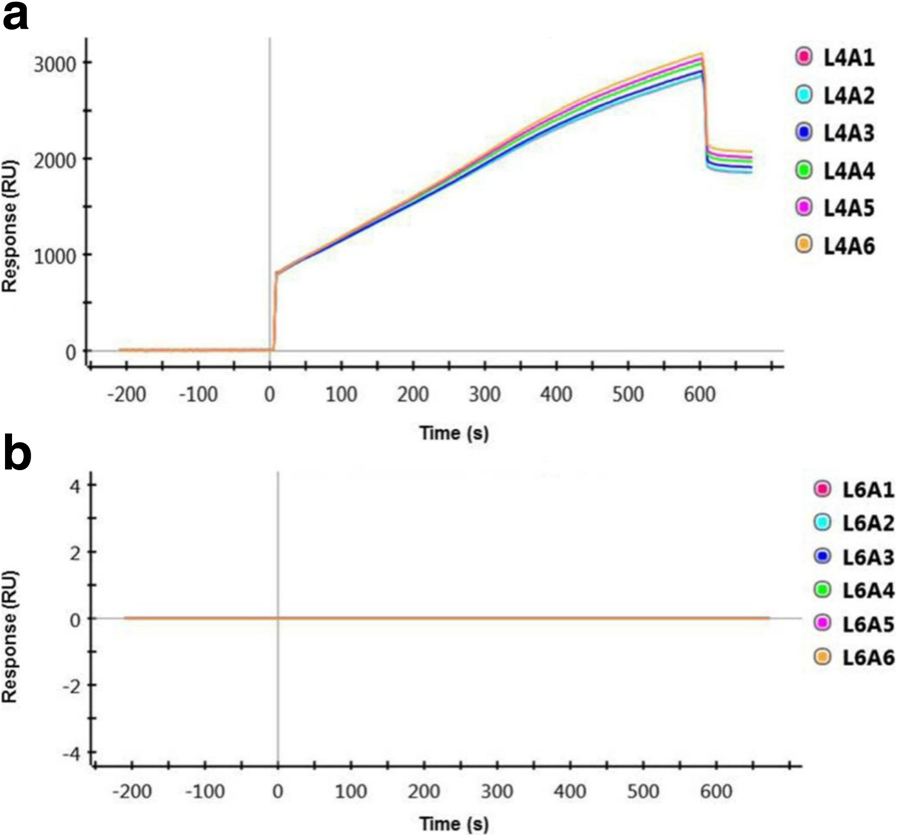
Smurf2 immobilisation. **a** Stable baseline SPR responses obtained from immobilisation of Smurf2 on the GLC chip surface. Following ligand injection, the channel L4 stably captured Smurf2 protein giving a response of ~ 2000 response units (RU). **b** L6 was subsequently used as a reference channel

Figure 4 gives the SPR responses obtained from channel L4 during Smurf2 immobilization (7.5 μg/ml). Channel L6 was used only as a reference channel, to offset buffer shift and subtract non-specific interactions of the analyte with the chip surface from SPR responses in the Smurf2 channel. In practice, a reference channel need not be immobilized with protein but can simply be NHS-activated and then blocked with ethanolamine to remove (negatively charged) carboxyl groups and reduce electrostatic interactions. Following immobilization, 1 M ethanolamine-HCl was used to deactivate the ligand channel and to block residual NHS-activated carboxyl groups, to remove loosely bound Smurf2 molecules and ensure a uniform baseline. A stable baseline response of ~ 2000 RU was obtained in channel L4 following Smurf2 capture, so there was no drift in SPR response that would affect the apparent kinetics during CNKSR2 interaction analysis.

#### CNKSR2-Smurf2 interactions- one-shot kinetics approach

The changes in refractive index occurred during the direct interaction between two proteins could be analysed by a novel method called Surface plasmon resonance (SPR). The ligand protein is immobilised on a sensor chip via covalent coupling. The analyte is injected over the chip surface and any binding between the two which results in a change in surface mass is recorded and measured as a change in refractive index. In the present study, we have examined the potential interaction between Smurf2 and CNKSR2**.** A unique feature of the ProteOn XPR36 system is its ability to collect kinetic data for all the four different analyte concentrations over the target ligand surface at one time.

**Fig. 5:**
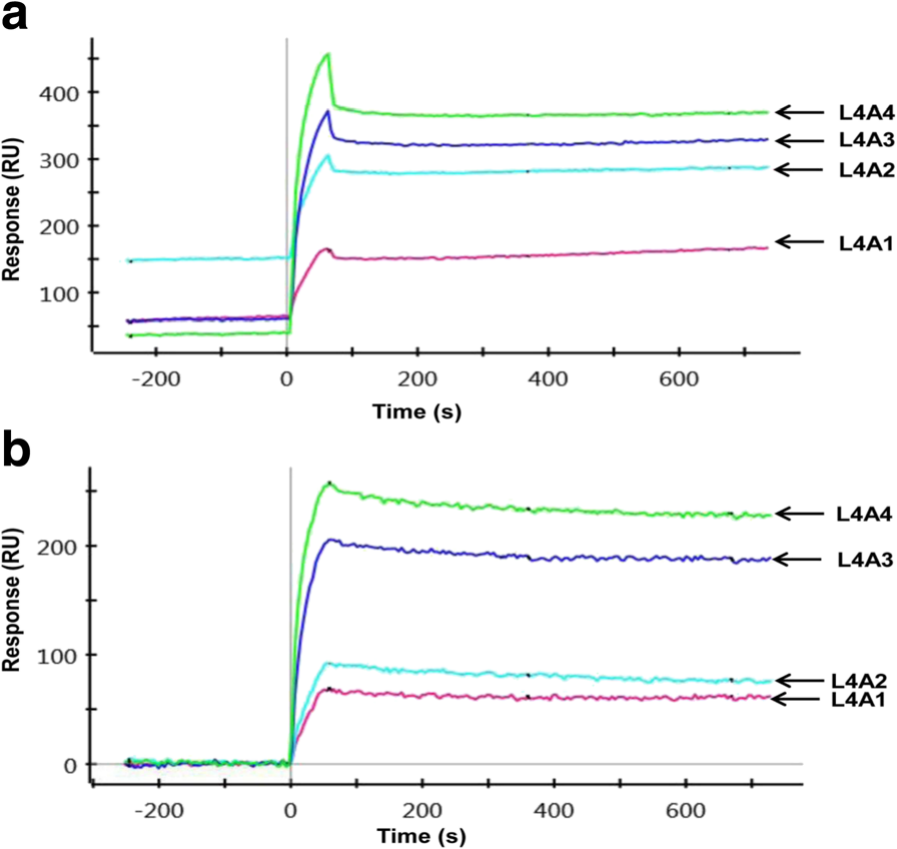
SPR Responses. **a** Raw data of Smurf2-CNKSR2 interaction responses for CNKSR2 concentrations from 0.25 μM (A1), 0.5 μM (A2), 1 μM (A3), and 2 μM (A4), injected over the Smurf2 channel (L4) on the ProteOn GLC chip. **b** Smurf2-CNKSR2 interaction responses after normalizing the baseline value and subtracting out data from the internal reference spots

Sensorgrams showing the raw and processed data (after subtraction of L6 responses) following the injection of the analyte CNKSR2 (injected for 60 s over the ligand surface) at four different concentrations from 0.25 μM, 0.5 μM, 1 μM, and 2 μM, in a two fold dilution series, immediately after the capture of Smurf2 are shown in Fig. 5a, b. The data from the reference spots were used to correct any baseline drift due to bulk refractive index change and non-specific bindings. The sensorgrams in the ligand channel overlap at each analyte concentration, showing good reproducibility.

#### Kinetic analysis

The SPR responses obtained from the interaction between different concentrations of CNKSR2 and Smurf2 were fitted using the Langmuir 1:1 bimolecular interaction model, according to the equation given below, using ProteOn Manager software version 3.1.0.6 (Bio-Rad, USA).

$$ \mathrm{A}+\mathrm{B}\underset{{\mathrm{k}}_{\mathrm{d}}}{\overset{{\mathrm{k}}_{\mathrm{a}}}{\rightleftarrows }}\kern0.5em \mathrm{AD} $$

**Fig. 6:**
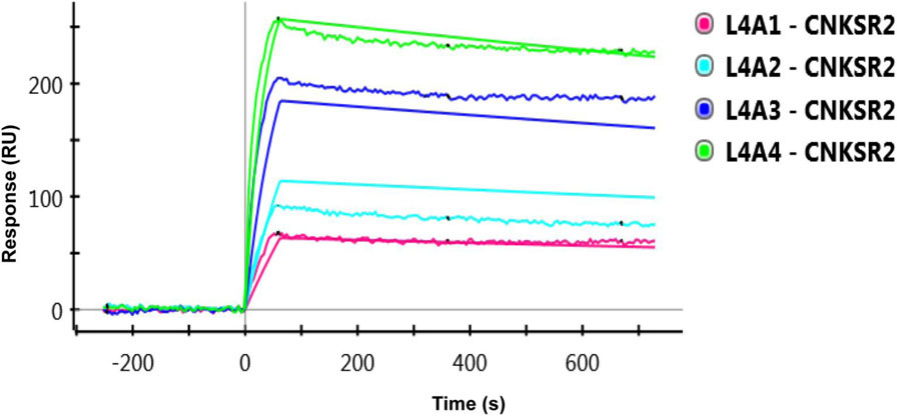
Curve fitting of Smurf2-CNKSR2 interaction responses using Langmuir model. Sensorgrams obtained from injections of CNKSR2 at concentrations of 0.25, 0.5, 1.0 and 2.0 μM over Smurf2 captured on the GLC chip surface. The curves depict the grouped fit of the data to a simple 1:1 bimolecular interaction model, yielding k_a_ = 1.52E + 04 M^− 1^ s^− 1^, k_d_ = 2.11E-04 s^− 1^ and K_D_ = 1.39E-08 M

Figure 6 shows the curve fits and the kinetic constants for the fits including single grouped k_a_ (association rate constant), k_d_ (dissociation rate constant), K_D_ (equilibrium or binding affinity constant) and R_max_ were obtained using the Langmuir model and are reported in Table 1. For the interaction surface, the modelled data overlay the experimental data, indicating that these binding events are well described by a simple interaction model.

**Table 1:** Kinetic constants for Smurf2-CNKSR2 interaction

Parameter	Concentration	k_a_	k_d_	K_D_	R_max_
Unit	M	1/Ms	1/s	M	RU
Scope	Local	Grouped	Grouped	Grouped	Grouped
Type	Constant	Fitted	Fitted	Calculated	Fitted
L4		1.52E + 04	2.11E-04	1.39E-08	304.21
L4A1	2.50E-07				
L4A2	5.00E-07				
L4A3	1.00E-06				
L4A4	2.00E-06				

The results from the Smurf2-CNKSR2 interaction seem to fit well using the Langmuir model in which one ligand molecule interacts with one analyte molecule. An equilibrium dissociation constant (K_D_) of 13.9 nM was calculated from the ratio of the association and dissociation rates indicating a stable interaction between Smurf2 and CNKSR2.

#### Depletion of Smurf2 decreased CNKSR2 protein stability with enhanced ubiquitination

**Fig. 7:**
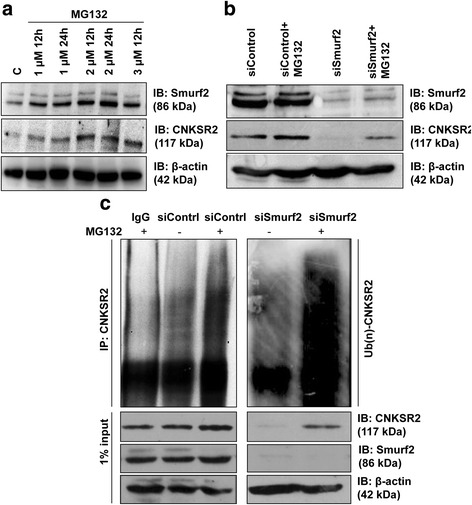
Depletion of Smurf2 accelerates degradation of CNKSR2. **a** Serum starved HEK293 cells were treated with MG132 at the indicated doses and time intervals and expression of CNKSR2 was assessed using western blot indicating proteasome mediated degradation of CNKSR2. **b** Decline in CNKSR2 expression induced by Smurf2 siRNA was rescued by treating the cells for 4 h with 10 μM MG132 44 h post-transfection. **c** Depletion of Smurf2 results in enhanced polyubiquitination and proteasomal degradation of CNKSR2. CNKSR2 was immunoprecipitated from MDA-MB-231 cells transfected with Smurf2 siRNA and control siRNA. Cells were treated for 4 h with 10 μM MG132 44 h post-transfection. The corresponding lysates were denatured and immunoprecipitated (IP) with rabbit CNKSR2 antibody, followed by Western blotting (IB) of the immune complexes with a mouse anti-ubiquitin antibody. The input proteins in cell lysates were also probed by the indicated antibodies

We reported previously that Smurf2 knockdown significantly downregulated the CNKSR2 protein levels in MDA-MB-231, MCF-7, SW480, and SCC131 cancer cell lines without any effect on CNKSR2 mRNA, suggesting that Smurf2 controls the CNKSR2 protein level possibly through proteolytic regulation. Likewise, a comparatively rapid degradation of CNKSR2 was observed in Smurf2 depleted cells following cycloheximide chase assay, implying the potential role of Smurf2 in regulating the stability of CNKSR2 protein [9]. Lending further support that Smurf2 modulates CNKSR2 expression, we analyzed the role of proteasome mediated degradation in maintaining the stability of CNKSR2 using the proteasome inhibitor, MG132. Our experiments showed increase in CNKSR2 expression following MG132 treatment. Concomitantly, the expression of Smurf2 was also found to be upregulated following MG132 treatment (Fig. 7a). Further we examined whether Smurf2 depletion accelerates the proteasome mediated degradation of CNKSR2. As we expected, treatment with MG132 substantially rescued the down-regulation of CNKSR2 in Smurf2 depleted cells (Fig. 7b). Correspondingly, to analyze whether the proteasome mediated degradation of CNKSR2 was dependent on polyubiquitination, CNKSR2 was immunoprecipitated from Smurf2-depleted MDA-MB-231 cells treated with MG132, and then analyzed by immunoblotting with mouse ubiquitin antibody (Fig. 7c). Polyubiquitination of CNKSR2 was significantly enhanced in cells treated with Smurf2 siRNA and MG132, compared with cells treated with non-specific siRNAs and MG132. Our results therefore suggest that depletion of Smurf2 leads to enhanced polyubiquitination and subsequent degradation of CNKSR2.

#### Smurf2 and CNKSR2 exhibit coordinated expression in human breast cancer progression model cell lines

**Fig. 8:**
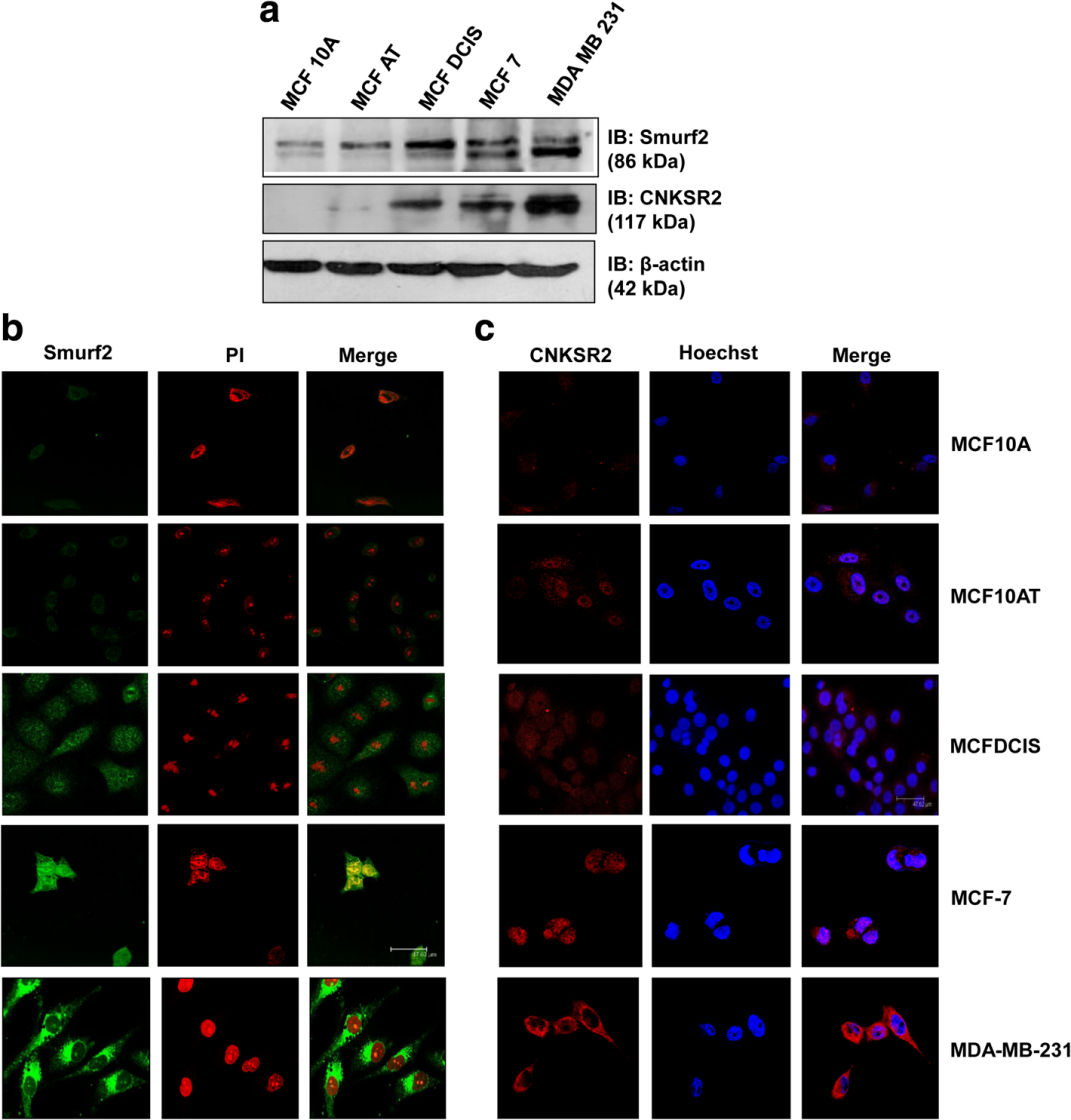
Smurf2 and CNKSR2 expression in an in vitro model of breast cancer progression. **a** Western blot analysis of Smurf2 and CNKSR2 expression in proliferating MCF10A cell line series; β-actin was used as a loading control. **b** Expression of Smurf2 and (**c**) CNKSR2 at the cellular level increases with tumorigenic potential of breast cancer cells and is localized in the nucleus and the cytoplasm (60× magnification)

Since the expression of Smurf2 was found to be elevated in human breast cancer cell lines and is involved in the proliferation and migration of breast cancer cells via interaction with CNKSR2, we investigated its role in the advancement of breast cancer by examining the expression along with CNKSR2 in an in vitro breast cancer progression model comprising MCF10A, MCF10AT, MCFDCIS, MCF-7 and MDA-MB-231. This series of cell lines provides a unique opportunity to study breast cancer progression, induced in a defined method, in a common cell background. Interestingly, we observed that both Smurf2 and CNKSR2 displayed a comparatively similar pattern of expression in mammary cells with increasing tumorigenic potential. Together Smurf2 and CNKSR2 was found to be significantly upregulated in MDA-MB-231 cells, even though a moderately high expression was also observed in MCFDCIS and MCF-7 cells compared to the normal (MCF10A) and premalignant (MCF10AT) cell lines (Fig. 8a).

Furthermore, subcellular localization of Smurf2 and CNKSR2 in breast progression model cells (Fig. 8b, c) also exhibited an analogous expression consistent with the western blot results showing progressively elevated expression with increasing tumorigenic potential. In normal and pre-maligant cells, Smurf2 and CNKSR2 were found to have a very low expression and are localized mainly in the nucleus. However, in MCFDCIS and MCF-7 we observed a more intense expression of Smurf2 and CNKSR2 in both the nucleus and cytoplasm. Alternatively, in MDA-MB-231 cells, both Smurf2 and CNKSR2 showed a significantly high expression localized mainly in the cytoplasm. Only a very mild expression of Smurf2 and CNKSR2 was observed in the nucleus.

#### Expression of Smurf2 and CNKSR2 is dysregulated in human breast tissues

**Fig. 9:**
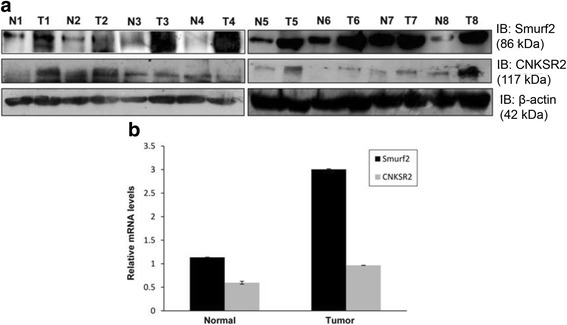
Expression of Smurf2 and CNKSR2 in primary breast tumors. **a** Lysates from paired samples of human primary breast tumors and adjacent normal breast tissues were analyzed for Smurf2 and CNKSR2 expression by western blotting. β-actin was used as the loading control. **b** Expression levels of Smurf2 and CNKSR2 mRNA in human primary breast tumors and normal tissues. GAPDH was used as the internal loading control

Recent reports by Jin et al.*,* 2009 highlighted the importance of Smurf2 proteins in breast cancer [14]; therefore to study the functional role of Smurf2 and CNKSR2 in human breast cancer tissues, we initially analyzed the expression levels of Smurf2 and CNKSR2 proteins in eight matched-sets of primary mammary carcinoma (Invasive Ductal Carcinoma, IDC) and adjacent normal breast tissue samples by western blotting. As shown in Fig. 9a, 87.5% of mammary carcinoma had elevated expression of Smurf2 compared to their normal counterparts. Similarly, CNKSR2 was also found to be overexpressed in 62.5% of malignant breast tumors compared to the adjacent normal breast tissues. We further analyzed the mRNA levels of Smurf2 and CNKSR2 in human breast cancer tissue samples (*n* = 8 for IDC) and adjacent normal breast tissue (*n* = 6 for normal) by real time quantitative PCR and observed approximately three fold upregulation in the expression of Smurf2 in invasive mammary carcinoma compared to non-tumor tissues (Fig. 9b). However, CNKSR2 does not exhibit a significant upregulation at the mRNA level in breast tumor samples compared to normal breast tissues (Fig. 9b), which indicate that expression status of Smurf2 and CNKSR2 is mainly associated at the protein level rather than at the mRNA level.

#### Smurf2 and CNKSR2 expression is associated with progressive changes in breast cancer

**Fig. 10:**
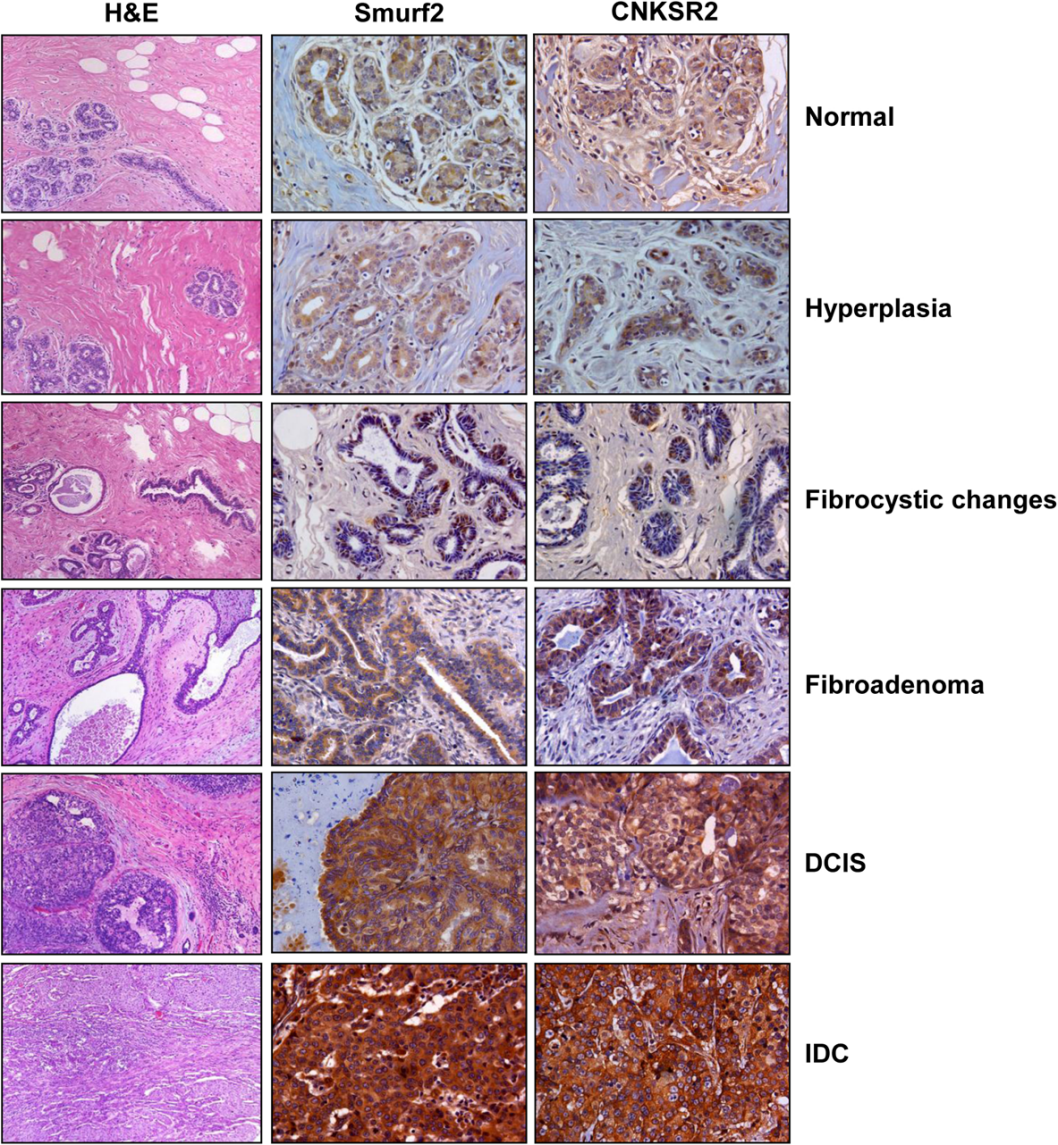
Smurf2 and CNKSR2 expression in normal, pre-invasive and invasive breast tissues. Both Smurf2 and CNKSR2 showed an increased expression with progressive changes in breast cancer. H & E images were adapted from Pantomics site

Notably high expression of Smurf2 in human breast cancer tissues and cell lines and its harmonized expression with CNKSR2 in breast cancer progression model cell lines prompted us to investigate the expression status of Smurf2 and CNKSR2 in human breast cancer subtypes by analyzing tissues from various stages of mammary carcinoma, including (in order of increasing malignant potential) normal, usual hyperplasia, fibrocystic changes, fibroadenoma, carcinoma-in-situ, and invasive ductal carcinoma (Fig. 10), thereby to explore the possible association between Smurf2 and CNKSR2 in progression of breast cancer and to translate our in vitro findings. The TMA included 8 cases of normal/hyperplasia, 7 cases each of fibrocystic changes, fibroadenoma, and ductal carcinoma in situ, and 55 cases of invasive ductal carcinoma.

As shown in Additional file 1: Table S1, Smurf2 expression was significantly associated with grade of the tumor (*P* < 0.001). Most of the normal/hyperplasia (87.5%), and fibrocystic (85.71%) tumors exhibited a very low expression of Smurf2. Only 1 out of the 7 fibroadenomas (14.29%) showed an intense expression of Smurf2 whereas, the remaining 6 samples (85.72%) had only mild or moderate expression of Smurf2; the same proportion as for the 7 DCIS cases. However, among the IDC cases, 33 out of the 55 IDC tumors (60.00%) dislayed a very high expression of Smurf2. Simultaneously, we also analyzed the expression levels of CNKSR2 in the same set of samples and observed a statistically significant association with tumor grade (*P* < 0.001). Most of the normal/hyperplasia (75.00%) and fibrocystic cases (57.14%) showed only a mild expression of CNKSR2, whereas the intensity of CNKSR2 expression increases with progression in tumor grade with 14.29% of fibroadenomas, 42.86% of DCIS and 52.73% of IDC’s showing intense expression of CNKSR2. Both Smurf2 and CNKSR2 showed a very low nuclear expression in normal luminal epithelial cells along with the cytoplasmic expression, whereas in higher grades of the tumors, the expression of Smurf2 and CNKSR2 was found to be exclusively localized in the cytoplasm. Altogether, the expression of Smurf2 and CNKSR2 displayed a statistically significant association with progressive grades of breast tumor samples as evidenced by Chi squared test.

In addition to Smurf2 and CNKSR2, we also analyzed the ER, PR, and HER2 status of the 84 representative cases, provided along with the tissue microarray data by Pantomics, and observed that only 14.29% of DCIS and 14.55% of IDC’s showed intense expression of ER compared to the normal and fibrocystic tumors (0.00%) (*P* < 0.001). Similar expression pattern was also observed for PR in normal and IDC cases (*P* < 0.05). However, the expression of HER2 was found to be significantly high in DCIS (57.14%) and IDC (41.82%) samples compared to the normal breast tissue samples (12.50%) (*P* < 0.05).

For further statistical analysis, we classified the total 84 breast tissue samples into two major groups (i) Normal/Benign or non-malignant (including normal/hyperplasia, fibrocystic changes and fibroadenoma, *n* = 22) and (ii) Malignant (including DCIS and IDC, *n* = 62) and on the basis of the staining intensity we classified the samples into two classes, (i) with No/Mild and (ii) with Moderate/Intense staining (Table 2). Our studies have shown that 56 out of 62 malignant tumors (90.32%) showed intense expression of Smurf2 whereas only 6 out of 22 non-malignant tumors (27.27%) showed high Smurf2 positivity (*P* < 0.001). Concomitantly, CNKSR2 expression also exhibited a similar pattern with 54 out of 62 malignant tumors (87.10%) showing moderate to intense expression of CNKSR2 compared with non-malignant samples, where only 5 out of 22 cases (22.73%) were found to have high expression of CNKSR2 (*P* < 0.001). However, the expression levels of ER and PR did not exhibit a statistically significant difference among non-malignant and malignant tumors (*P* = 0.875, *P* < 0.05) whereas HER2 was found to be intensely expressed in 35 out of 62 malignant tumors (56.45%) compared to 6 out of 22 non-mlaignant tumors (27.27%) showing a statistically significant association (*P* < 0.05).

**Table 2:** Expression analysis of Smurf2, CNKSR2, ER, PR and HER2 in non-malignant and malignant breast tissue samples

		Grade of Tumour			
Proteins studied	Staining intensity	Non-Malignant (*n* = 22)	Malignant (*n* = 62)	Total (*n* = 84)	*P*-Value
Smurf2	No/Mild	16(72.73%)	6(9.68%)	22(26.19%)	P < 0.001
	Moderate/Intense	6(27.27%)	56(90.32%)	62(73.81%)	
CNKSR2	No/Mild	17(77.27%)	8(12.90%)	25(29.76%)	P < 0.001
	Moderate/Intense	5(22.73%)	54(87.10%)	59(70.24%)	
ER	No/Mild	16(72.73%)	44(70.97%)	60(71.43%)	P = 0.875
	Moderate/Intense	6(27.27%)	18(29.03%)	24(28.57%)	
PR	No/Mild	10(45.45%)	45(72.58%)	55(65.48%)	P < 0.05
	Moderate/Intense	12(54.55%)	17(27.42%)	29(34.52%)	
HER2	No/Mild	16(72.73%)	27(43.55%)	43(51.19%)	P < 0.05
	Moderate/Intense	6(27.27%)	35(56.45%)	41(48.81%)	

Further we analyzed the association between Smurf2, CNKSR2, ER, PR, and HER2 expression with non-malignant and malignant tumors using multiple logistic regression analysis taking non-malignant tumors as the reference (Table 3). We observed that, malignant tumors were 24.88 times more likely to exhibit moderate/intense expression of Smurf2, when non-malignant tumor was the reference (OR =24.88, 95% CI: 7.05–87.79). Moreover we calculated the adjusted Odds ratio to study the actual effect of a single factor when all other factors were kept constant. According to the adjusted Odds ratio, malignant tumors were 25.94 times more likely to exhibit moderate/intense expression of Smurf2, taking non-malignant tumor as the reference (OR = 25.94, 95%CI: 3.86–174.06). Similarly, considering the unadjusted and adjusted Odds ratios of CNKSR2 expression, malignant tumors were 22.95 and 16.88 times more likely to exhibit moderate/intense expression of CNKSR2, when non-malignant tumor is the reference (unadjusted OR =22.95, 95% CI: 6.62–79.56; adjusted OR = 16.88, 95% CI: 2.64–107.82). Contrarily, ER and PR did not exhibit a statistically significant expression in malignant tumors considering non-malignant tumors as the reference. However, HER2 expression showed a statistically significant difference between non-malignant and malignant tumors. In malignant tumors there was 3.45 times higher chance for observing moderate/intense expression of HER2 when non-malignant tumors were taken as the reference (unadjusted OR =3.45, 95% CI: 1.19–10.01; adjusted OR = 2.77, 95% CI: 0.57–13.4).

**Table 3:** Multiple logistic regression analysis: Significant association between Smurf2 and CNKSR2 expression with histopathological grade of the tumor

Proteins	Tumor grade with staining intensity	OR	95% CI	OR*	95% CI*
Smurf2	No/Mild	1		1	
	Moderate/Intense	24.88	7.05–87.79	25.94	3.86–174.06
CNKSR2	No/Mild	1		1	
	Moderate/Intense	22.95	6.62–79.56	16.88	2.64–107.82
ER	No/Mild	1		1	
	Moderate/Intense	1.09	0.36–3.23	0.13	0.01–1.33
PR	No/Mild	1		1	
	Moderate/Intense	0.31	0.11–0.86	0.33	0.05–1.99
HER2	No/Mild	1		1	
	Moderate/Intense	3.45	1.19–10.01	2.77	0.57–13.4

*OR and *CI represents adjusted Odds ratio and adjusted 95% CI

OR and CI represents unadjusted Odds ration and unadjusted 95% CI

Eventhough our descriptive analysis indicated a statistically significant association between Smurf2 and CNKSR2 with progressive breast tumor cases, we further analyzed the association between expression status of Smurf2 relative to CNKSR2 in non-malignant and malignant tumors (Table 4). Interestingly, among malignant tumors, 51 out of 62 cases (82.26%) showed moderate/intense expression of CNKSR2 when Smurf2 expression was moderate/intense. Only 5 out of 62 malignant tumors (8.06%) showed a no/mild expression of CNKSR2 when Smurf2 expression was moderate/intense. Simultaneously, among non-malignant tumors, 13 out of 22 cases (59.09%) showed no/mild expression of CNKSR2 when Smurf2 expression was no/mild. Only 3 out of 22 non-malignant tumors (13.64%) showed a moderate/intense expression of CNKSR2 when Smurf2 expression was no/mild. Overall, the expression pattern of Smurf2 and CNKSR2 showed a significant positive association (*P* < 0.001) between each other among non-malignant and malignant tumors.

**Table 4:** Expression of Smurf2 relative to CNKSR2 in non-malignant and malignant breast tumors

		Non-Malignant	Malignant	*P*-value
		(*n* = 22)	(*n* = 62)	
Smurf2	CNKSR2			
no/mild	no/mild	13(59.09%)	3(4.84%)	< 0.001
no/mild	mod/intense	3(13.64%)	3(4.84%)	
mod/intense	no/mild	4(18.18%)	5(8.06%)	
mod/intense	mod/intense	2(9.09%)	51(82.26%)	

#### Smurf2 and CNKSR2 expression is associated with the ER, PR, and HER2 status of breast tissue samples

We next observed that the expression levels of Smurf2 and CNKSR2 in relation with the hormonal (ER and PR) and HER2 status of breast tissue samples showed a statistically significant difference among non-malignant and malignant tumors (Tables 5 and 6). Our studies have shown that when ER was no/mild, 61.29 and 58.06% of malignant tumors showed moderate/intense expression of Smurf2 and CNKSR2, while only 29.03% of malignant tumors showed moderate/intense expression of Smurf2 and CNKSR2 when ER was moderate/intense. Similarly, when PR was no/mild, 66.13 and 58.06% of malignant tumors showed moderate/intense expression of Smurf2 and CNKSR2. However, when PR was moderate/intense, only 24.19 and 29.03% of malignant tumors showed moderate/intense expression of Smurf2 and CNKSR2. Contrarily, we observed that when HER2 was no/mild, only 40.32 and 38.71% of malignant tumors showed moderate/intense expression of Smurf2 and CNKSR2 whereas 50.00 and 48.39% of malignant tumors showed moderate/intense expression of Smurf2 and CNKSR2 when HER2 was moderate/intense. Overall, when the expression of ER, and PR was low, the expression of Smurf2 and CNKSR2 was high, whereas Smurf2 and CNKSR2 showed a comparatively high expression when HER2 expression was high.

**Table 5:** Expression of Smurf2 relative to ER, PR and HER2 status in non-malignant and malignant breast tumors

		Non-Malignant	Malignant	*P*-value
		(*n* = 22)	(*n* = 62)	
Smurf2	ER			
no/mild	no/mild	14(63.64%)	6(9.68%)	< 0.001
no/mild	mod/intense	2(9.09%)	0(0.00%)	
mod/intense	no/mild	2(9.09%)	38(61.29%)	
mod/intense	mod/intense	4(18.18%)	18(29.03%)	
Smurf2	PR			
no/mild	no/mild	9(40.91%)	4(6.45%)	< 0.001
no/mild	mod/intense	7(31.82%)	2(3.23%)	
mod/intense	no/mild	1(4.55%)	41(66.13%)	
mod/intense	mod/intense	5(22.73%)	15(24.19%)	
Smurf2	HER2			
no/mild	no/mild	12(54.55%)	2(3.23%)	< 0.001
no/mild	mod/intense	4(18.18%)	4(6.45%)	
mod/intense	no/mild	4(18.18%)	25(40.32%)	
mod/intense	mod/intense	2(9.09%)	31(50.00%)

**Table 6:** Expression of CNKSR2 relative to ER, PR and HER2 status in non-malignant and malignant breast tumors

		Non-Malignant	Malignant	*P*-value
		(*n* = 22)	(*n* = 62)	
CNKSR2	ER			
no/mild	no/mild	14(63.64%)	8(12.9%)	< 0.001
no/mild	mod/intense	3(13.64%)	0(0.00%)	
mod/intense	no/mild	2(9.09%)	36(58.06%)	
mod/intense	mod/intense	3(13.64%)	18(29.03%)	
CNKSR2	PR			
no/mild	no/mild	14(63.64%)	8(12.9%)	< 0.001
no/mild	mod/intense	3(13.64%)	0(0.00%)	
mod/intense	no/mild	2(9.09%)	36(58.06%)	
mod/intense	mod/intense	3(13.64%)	18(29.03%)	
CNKSR2	HER2			
no/mild	no/mild	14(63.64%)	3(4.84%)	< 0.001
no/mild	mod/intense	3(13.64%)	5(8.06%)	
mod/intense	no/mild	2(9.09%)	24(38.71%)	
mod/intense	mod/intense	3(13.64%)	30(48.39%)	

#### Stable knockdown of Smurf2 expression restrained tumorigenic potential of breast cancer cells in a CNKSR2 dependent manner

**Fig. 11:**
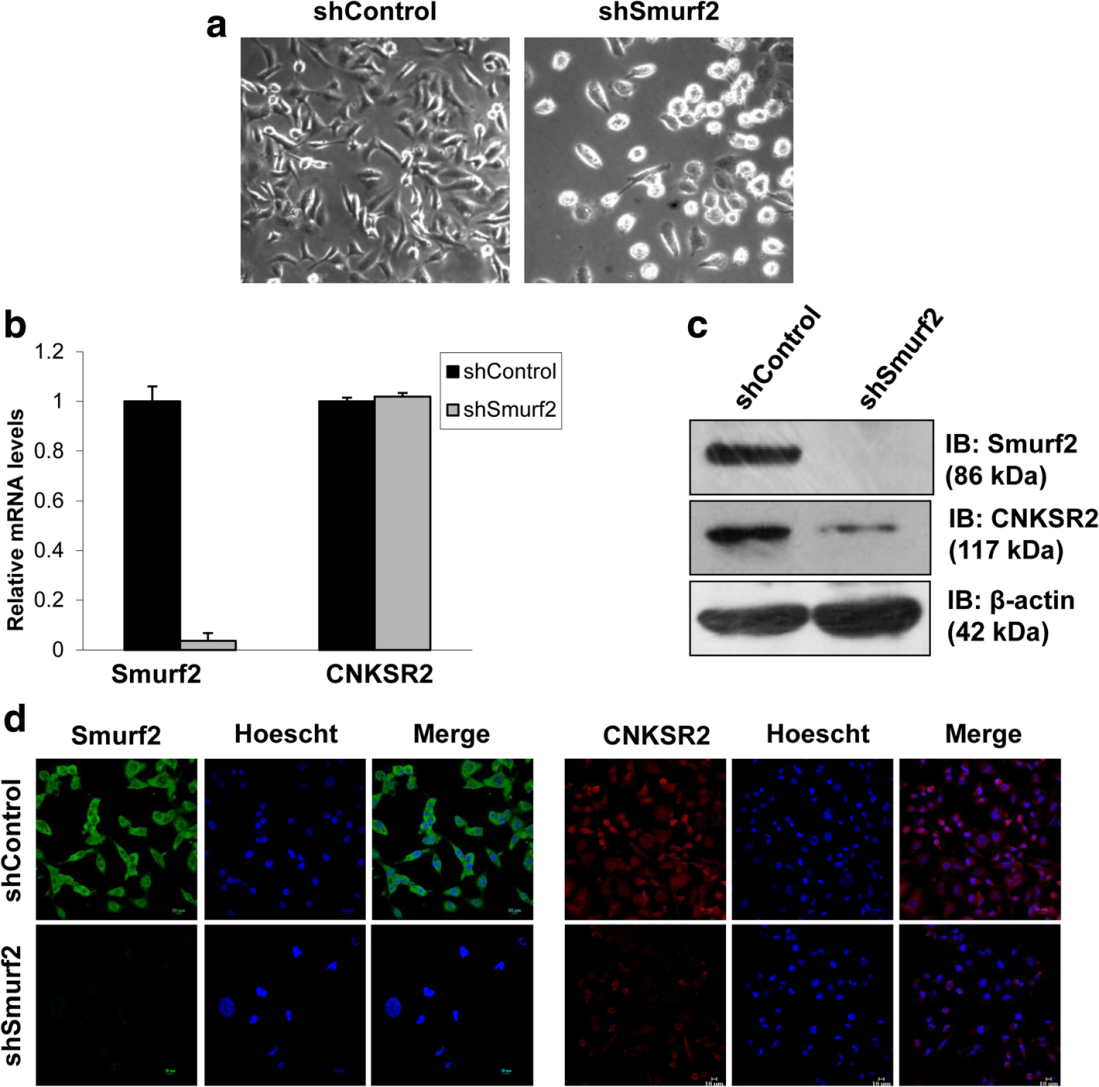
Knockdown of Smurf2 decreased the level of endogenous CNKSR2. **a** Representative images of MDA-MB-231 cells expressing either control or Smurf2 shRNA constructs. **b** qRT-PCR analysis of Smurf2 shRNA knockdown cells indicated that Smurf2 depletion had little to no effect on CNKSR2 mRNA levels. (**c**, **d**) Depletion of Smurf2 leads to decreased CNKSR2 expression in MDA-MB-231cells. Results represented as mean ± standard deviation from three independent experiments

We reported previously that transient inhibition of Smurf2 expression decreased the proliferative potential of breast cancer cells by modulating CNKSR2 mediated PI3K-AKT signaling csascade [9]. In order to further ascertain the effect of Smurf2 knockdown on the proliferative potential of breast cancer cells, we developed stable lentiviral Smurf2 short hairpin RNA (shRNA) knockdown clones in MDA-MB-231 cell line. The downregulation of Smurf2 was confirmed by qRT-PCR, western blot and immunofluorescence (Fig. 11b, c, d). More than 90% knockdown in the levels of Smurf2 was observed in the Smurf2 shRNA clones (shSmurf2) compared to the non-target control shRNA-expressing clones (shControl). Concomitantly, the protein level expression of CNKSR2 was also found to be significantly downregulated in Smurf2 knockdown cells compared to the control cells (Fig. 11c, d). However, the CNKSR2 mRNA levels remained relatively unchanged after Smurf2 knockdown (Fig. 11b). Altogether, these data suggest that Smurf2 positively regulates CNKSR2 expression at the post-transcriptional level. Furthermore we observed that Smurf2 knockdown significantly decreases the tumorigenic potential of breast cancer cells [9]. All these results suggest that Smurf2 plays a key role in regulating the tumorigenic properties of breast cancer cells in a CNKS2 dependent manner.

## Discussion

Smurf2 E3 ubiquitin ligases plays crucial physiological roles in regulating multiple cellular processes including TGF-β/BMP signaling, cell polarity, cell migration, and mitotic regulation by specifically targeting corresponding cellular substrates for ubiquitination which can either lead to stabilization and or proteasome mediated degradation [15–18]. In the present study we have demonstrated the novel regulation of the multi-functional scaffold protein CNKSR2 by the WW-HECT protein Smurf2. Consistent with the presence of the Smurf2-interacting PY motif in CNKSR2, Smurf2 was found to be immunoprecipitated with CNKSR2 and vice versa, which demonstrated a direct physical interaction between CNKSR2 and Smurf2 (Fig. 1). Interestingly, we observed that though CNKSR2 interacts with Smurf2, it does not bind to Smurf1 (Fig. 1a), which also belong to the Nedd4 family of E3 ubiquitin ligases possessing WW domains in its structure indicating specific Smurfs can recognize CNKSR2 specifically, which also provide evidence of CNKSR2 as a novel class of scaffold proteins regulated by Smurf2.

Functional activity and accessibility of Smurfs towards different substrates is mainly regulated by its subcellular localization [5, 10]. As shown in Fig. 3 we observed that both Smurf2 and CNKSR2 co-localizes particularly in the cytoplasm, eventhough a recognizable expression of Smurf2 was also observed in the nucleus. Because Smurf2 localization is highly dynamic and as previously reported Smurf2 interacts with TGF-β receptor in distinct endosomal compartments [19], we hypothesize that Smurf2 may be interacting mainly with the cytoplasmic CNKSR2 pool which may further reinforce the physical interaction between Smurf2 and CNKSR2, leading to its increased stability.

In order to further confirm the physical interaction between Smurf2 and CNKSR2 and to measure the kinetics of interaction, we have described for the first time, a simple and rapid, label-free, real-time, multiplex surface plasmon resonance (SPR) assay [20, 21] by which Smurf2-CNKSR2 interactions could be studied. We observed that, the results for Smurf2-CNKSR2 interaction fit well with a Langmuir model and give K_D_ values that indicate a high affinity, and stable interaction between Smurf2 and CNKSR2 (Fig. 6 and Table 1). This is the first time, to our knowledge, that real-time Smurf2-CNKSR2 interactions have been demonstrated using SPR. We believe this approach could be extended to study the effect of a variety of other factors on the interaction.

Protein ubiquitylation can control signaling pathways by regulating the cellular levels of a protein, by controlling its subcellular localization, by preventing accumulation of defective or damaged proteins, and in some instances, by preventing protein degradation [22]. Together with, our studies explored the consequence of the physical interaction between Smurf2 and CNKSR2, which indicated the presence of a ubiquitinated CNKSR2 in the presence of wild type Smurf2 compared with ligase deficient Smurf2 (Fig. 2c). However, Smurf2 mediated ubiquitination of CNKSR2 leads to stabilization of CNKSR2 instead of degradation as indicated by increased expression of CNKSR2 in Smurf2WT transfected cells. Unlike the interaction of Smurf2 with Mad2 [23] and EGFR [24], we observed that CNKSR2 can interact with both wild type and ligase inactive mutant of Smurf2. However, the expression of endogenous CNKSR2 was found to be significantly upregulated in Smurf2WT transfected cells (Fig. 2a, b) which may be due to the editing of CNKSR2 ubiquitination by Smurf2WT, leading to increased stabilization of CNKSR2 expression. Furthermore, we demonstrated that the loss of Smurf2 caused rapid CNKSR2 degradation mediated by enhanced polyubiquitination and proteasomal degradation (Fig. 7e).

The mechanism by which Smurf2 controls CNKSR2 stability remains to be elucidated. We hypothesized that regulation of CNKSR2 by Smurf2 occurs through diverse mechanisms. Smurf2-CNKSR2 interaction exhibit a significantly high binding affinity as observed during SPR analysis and further leads to ubiquitination, which might lead to increased stability of CNKSR2 in Smurf2 overexpressed cells. Eventhough the ubiquitination of CNKSR2 by Smurf2 may increase the stability of CNKSR2; Smurf2 may target an intermediary E3 ligase for degradation to stabilize CNKSR2. This might be responsible for the comparatively increased expression of CNKSR2 in ligase deficient Smurf2C716A transfected cells also compared to the control plasmid transfected cells (Fig. 2b). Consistent with the ligase-independent function of Smurf2 is a previous report that overexpression of wild-type or ligase dead Smurf2 induces senescence [25]. In addition, Smurf2 may sequester CNKSR2 away from cellular locations where it could encounter its E3 ligase. Alternatively, Smurf2 may instead mask regulatory epitopes for ubiquitination by other E3 ubiquitin ligases. On the other hand, Smurf2 may serve as an adaptor for an unidentified regulator that counteracts with another E3 ligase promoting CNKSR2 degradation.

Because Smurf2 and CNKSR2 were found to be involved in regulating the proliferation and invasion of breast cancer cells [9], we next examined the functional association of Smurf2 and CNKSR2 in the progression of breast cancer by analyzing the cellular level expression using MCF10 breast cancer progression model cell lines. Interestingly, we observed that both Smurf2 and CNKSR2 showed an integrated expression in breast cells with increasing tumorigenic potential (Fig. 8). Eventhough it is reported that Smurf2 is highly expressed in breast cancer [14], clinical information regarding the frequency and level of Smurf2 and CNKSR2 expression in breast cancer is incomplete, especially as regards differentiating the importance of Smurf2 and CNKSR2 expression in breast cancer subtypes with increasing tumorigenic potential including normal, usual hyperplasia, fibrocystic changes, fibroadenoma, carcinoma-in-situ (DCIS), and invasive ductal carcinoma (IDC) and its association with the ER, PR, and HER2 status of the breast tissue samples.

Using tissue microarray, which permits rapid high-throughput immunohistochemical analysis of several breast tissue samples simultaneously [26], Smurf2 and CNKSR2 expression was analyzed in breast cancers as well as in pre-invasive, and pre-neoplastic breast lesions and observed that our results extend the limited findings in previous reports [14]. Concomitantly, we observed that expression of Smurf2 and CNKSR2 was associated with stage of breast tumor progression, showing increased expression from normal, hyperplastic breast samples, and fibroadenoma to in situ carcinoma to invasive breast carcinoma (Fig. 10). Eventhough we found a statistically significant independent association between Smurf2 and CNKSR2 expression with progressive breast tumor cases by means of descriptive analysis and logistic regression analysis, we further studied the association between expression status of Smurf2 relative to CNKSR2 in non-malignant (Normal/Benign) and malignant tumors (in situ and invasive breast carcinoma) (Additional file 1: Table S1, Tables 2 and 3). Despite the independent association between Smurf2 and CNKSR2 expression with progressive breast tumor stages, the expression pattern of Smurf2 and CNKSR2 showed a significant positive association (*P* < 0.001) between each other among non-malignant and malignant tumors (Table 4). These findings agree with the putative role of Smurf2 and CNKSR2 in proliferation and invasiveness of breast cancer.

Breast cancer is a heterogeneous disease with a wide spectrum of clinical, pathologic, and molecular features [27, 28]. Recently, Liu et al.*,* [29] reported that expression of Smurf2 is associated with the ER, PR, and HER2 status of breast tumors, which instigated us to analyze the crosstalk between Smurf2 and CNKSR2 expression with the hormonal (ER and PR) and HER2 status of breast tissue samples. Interestingly, we observed a statistically significant association between Smurf2 and CNKSR2 expression with the ER, PR, and HER2 status of non-malignant and malignant tumors (Tables 5 and 6). Overall, we observed a comparatively high expression of Smurf2 and CNKSR2 when the expression of ER and PR was low, and HER2 was high. Importantly, Smurf2 and CNKSR2 expression has been associated with features of tumor aggressivity, ER and PR negativity and HER2 overexpression.

In order to assess the physiological significance of Smurf2-CNKSR2 interaction, we stably knocked down Smurf2 using Smurf2 shRNA in MDA-MB-231 cells, and observed that Smurf2 knockdown induced CNKSR2 degradation (Fig. 11), which leads to reduced pro-proliferative and tumorigenic properties of breast cancer cells.

## Conclusions

In summary, we illustrate a novel and specific interaction between Smurf2 and CNKSR2 which expand the list of substrates/interacting partners of Smurf2 E3 ubiquitin ligases by demonstrating that Smurf2 ubiquitinates, stabilizes, and positively regulates CNKSR2. However, the specific domain(s) involved in this interaction remains to be studied in detail. These results adopt CNKSR2 as a new family of targets by the Smurf E3 ubiquitin ligases and Smurfs as a new group of ubiquitin ligases that regulate CNKSR2 scaffold proteins. The interaction and stabilization of CNKSR2 are quite specific for the Smurf family, as they are highly preferred by Smurf2 rather than the closely related homolog, Smurf1 (Fig. 1). As reported previously [30] our observations have both basic as well as significant clinical relevance: (a) it identifies a molecular regulator, which may be critical in breast cancer cell–specific CNKSR2 overexpression (b) it also identifies Smurf2 as a novel therapeutic target, down-regulation of which can degrade CNKSR2 protein leading to reduced proliferation of breast cancer cells. Altogether, these findings motivate the investigation of the therapeutic efficacy of Smurf2 knockdown in treating CNKSR2-addicted cancers more effectively either as an individual therapy or in combination with already existing chemotherapy and/or radiotherapy.

## Additional file

Additional file 1: Smurf2, CNKSR2, ER, PR and HER2 expression in normal, pre-invasive and invasive breast tissue samples. (DOC 31 kb)

## Abbreviations

BMP: Bone morphologenetic proteins
CNKSR2: Connector enhancer of kinase suppressor of ras 2
HECT: Homologous to E6AP carboxyl terminus
PI3K: Phosphoinositide 3-kinase
Smurf2: Smad ubiquitin regulatory factor 2
TGF-β: Transforming growth factor β
