# Supplementary material for: Regulation of CNKSR2 protein stability by the HECT E3 ubiquitin ligase Smurf2, and its role in breast cancer progression
Source: BMC Cancer. 2018 Mar 13;18:284. doi: 10.1186/s12885-018-4188-x (PMC5850909; doi:10.1186/s12885-018-4188-x)
Supplement: Supplementary file 1 — Smurf2, CNKSR2, ER, PR and HER2 expression in normal, pre-invasive and invasive breast tissue samples. (DOC 31 kb) [file 12885_2018_4188_MOESM1_ESM.doc]

**Table S1: Smurf2, CNKSR2, ER, PR and HER2 expression in normal, pre-invasive and invasive breast tissue samples**

**Types of Tumor**

**Proteins Staining Normal/ Fibrocystic Fibroadenoma DCIS IDC Total P-value**

**studied intensity Hyperplasia changes (n=7) (n=7) (n=55) (n=84)**

**(n=8) (n=7)**

No 2(25.00%) 2(28.57%) 0(0.00%) 0(0.00%) 1(1.82%) 5(5.95%)

**Smurf2**  Mild 5(62.50%) 4(57.14%) 3(42.86%) 2(28.57%) 3(5.45%) 17(20.24%) P<0.001 Moderate 1(12.50%) 1(14.29%) 3(42.86%) 4(57.14%) 18(32.73%) 27(32.14%)

Intense 0(0.00%) 0(0.00%) 1(14.29%) 1(14.29%) 33(60.00%) 35(41.67%)

No 2(25.00%) 1(14.29%) 1(14.29%) 0(0.00%) 1(1.82%) 5(5.95%)

**CNKSR2** Mild 6(75.00%) 4(57.14%) 3(42.86%) 1(14.29%) 6(10.91%) 20(23.81%) P<0.001

Moderate 0(0.00%) 2(28.57%) 2(28.57%) 3(42.86%) 19(34.55%) 26(30.95%)

Intense 0(0.00%) 0(0.00%) 1(14.29%) 3(42.86%) 29(52.73%) 33(39.29%)

No 1(12.50%) 2(28.57%) 0(0.00%) 2(28.57%) 27(49.09%) 32(38.10%)

**ER** Mild 7(87.50%) 5(71.43%) 1(14.29%) 1(14.29%) 14(25.45%) 28(33.33%) P<0.001

Moderate 0(0.00%) 0(0.00%) 4(57.14%) 3(42.86%) 6(10.91%) 13(15.48%)

Intense 0(0.00%) 0(0.00%) 2(28.57%) 1(14.29%) 8(14.55%) 11(13.10%)

No 0(0.00%) 2(28.57%) 0(0.00%) 3(42.86%) 25(45.45%) 30(35.71%)

**PR** Mild 4(50.00%) 3(42.86%) 1(14.29%) 1(14.29%) 16(29.09%) 25(29.76%) P<0.05

Moderate 3(37.50%) 1(14.29%) 3(42.86%) 3(42.86%) 6(10.91%) 16(19.05%)

Intense 1(12.50%) 1(14.29%) 3(42.86%) 0(0.00%) 8(14.55%) 13(15.48%)

No 1(12.50%) 1(14.29%) 0(0.00%) 0(0.00%) 11(20.00%) 13(15.48%)

**HER2** Mild 6(75.00%) 4(57.14%) 4(57.14%) 1(14.29%) 15(27.27%) 30(35.71%) P<0.05

Moderate 0(0.00%) 1(14.29%) 3(42.86%) 2(28.57%) 6(10.91%) 12(14.29%)

Intense 1(12.50%) 1(14.29%) 0(0.00%) 4(57.14%) 23(41.82%) 29(34.52%)
